# Supplementary material for: Chemogenetic silencing reveals presynaptic Gi/o protein-mediated inhibition of developing hippocampal synchrony in vivo
Source: iScience. 2024 Sep 20;27(10):110997. doi: 10.1016/j.isci.2024.110997 (PMC11489827; doi:10.1016/j.isci.2024.110997)
Supplement: Document S1. Figures S1–S3 and Table S1 [file mmc1.pdf]

iScience, Volume *27*

Supplemental information

Chemogenetic silencing reveals presynaptic $G_{i/o}$ protein-mediated inhibition of developing hippocampal synchrony *in vivo*

Jürgen Graf, Arash Samiee, Tom Flossmann, Knut Holthoff, and Knut Kirmse

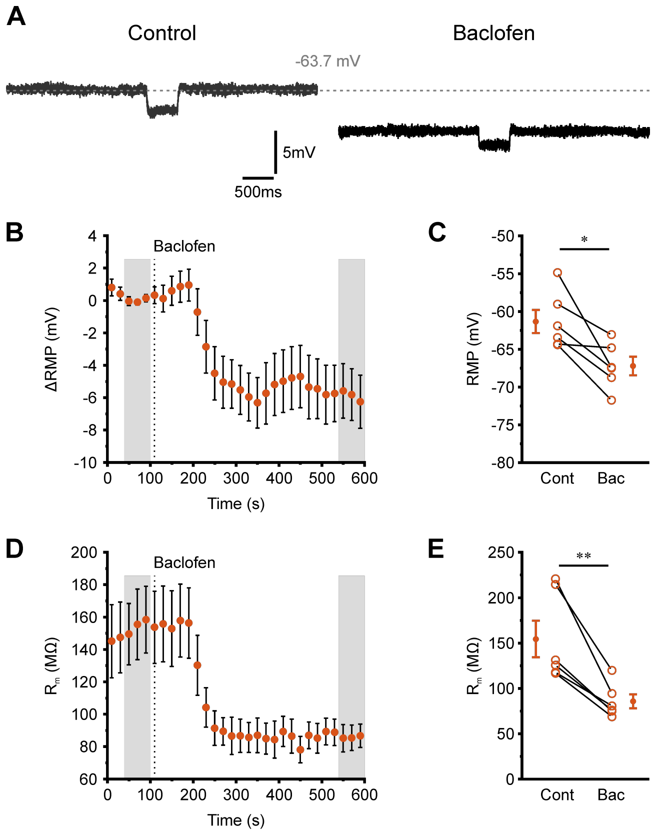

Figure S1. GABA$_B$R-mediated hyperpolarization in CA1 pyramidal cells at P29–31, related to Figure 2.

(A) Sample current-clamp recording ($I = 0$) of membrane potential before (*left*) and during (*right*) bath-application of the GABA$_B$R agonist baclofen (10 µM). Brief test pulses were used to estimate membrane resistance.

(B) Time-course of the change in resting membrane potential (ΔRMP) before and during bath-application of baclofen. The dotted line indicates the switch of solutions. Shaded areas indicate data points used for averaging and statistics.

(C) Quantification of RMP.

(D) Time-course of membrane resistance (R_m) before and during bath-application of baclofen. The dotted line indicates the switch of solutions. Shaded areas indicate data points used for averaging and statistics.

(E) Quantification of R_m.

Open symbols represent individual cells. Population data (closed symbols) are presented as mean ± SEM. $^{**}P<0.01$, $^{*}P<0.05$ (paired t-tests). See also Table S1.

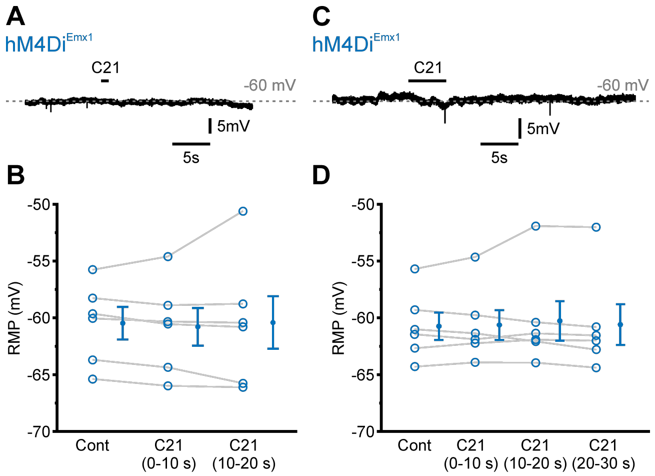

Figure S2. Membrane potential is unaffected by fast puff-application of C21 in CA1 pyramidal cells of *hM4Di*^{Emx1} mice at P13, related to Figure 2.

(A) Sample current-clamp recording ($I = 0$) of membrane potential before and after puff-application of C21 (10 μM) for 1 s.

(B) Quantification of RMP for a puff duration of 1 s. Median membrane potential was measured in 10-s-long non-overlapping bins.

(C) Sample current-clamp recording ($I = 0$) of membrane potential before and after puff-application of C21 (10 μM) for 5 s.

(D) Quantification of RMP for a puff duration of 5 s. Median membrane potential was measured in 10-s-long non-overlapping bins.

Open symbols represent individual cells. Population data (closed symbols) are presented as mean ± SEM. See also Table S1.

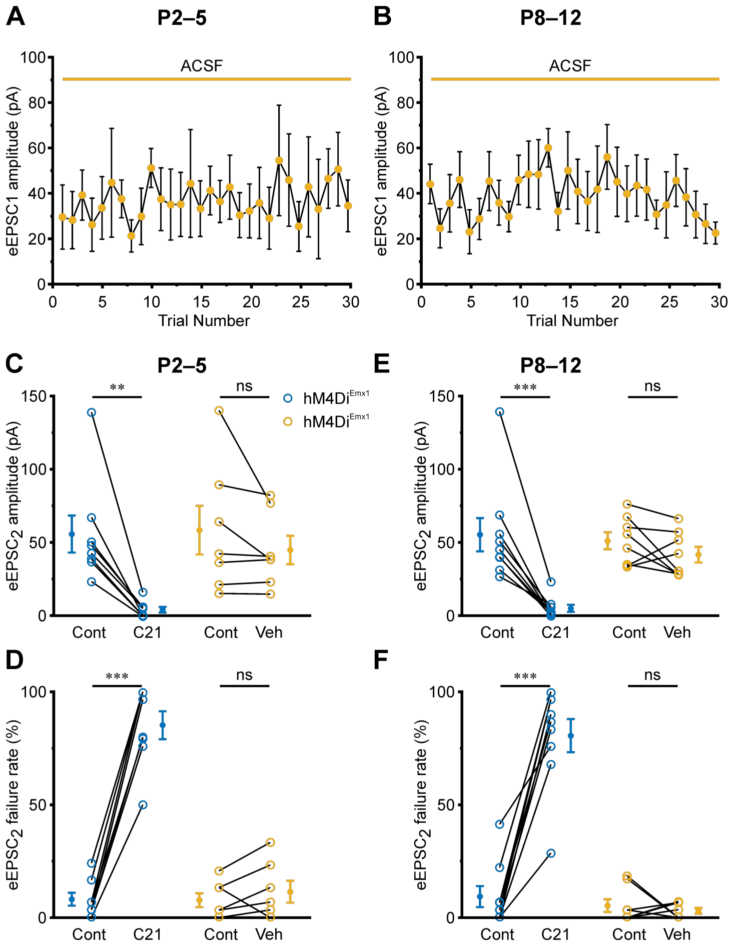

Figure S3. Stability of eEPSC amplitudes and C21 effects on eEPSCs, related to STAR Methods and Figure 5.

(A–B) $eEPSC_1$ amplitudes *versus* trial number at P2–5 (A) and P8–12 (B). Paired-pulse stimulation was performed at an inter-trial interval of 10 s. After trials #10 and #20, recordings were briefly interrupted to monitor access resistance. Data were obtained in standard ACSF (P2–5: n = 7 cells from four *hM4Di*^{Emx1} mice; P8–12: n = 8 cells from four *hM4Di*^{Emx1} mice).

(C–D) Mean amplitudes (C) and failure rates (D) of EPSCs evoked by the second pulse ($eEPSC_2$) at P2–5.

(E–F) Mean amplitudes (C) and failure rates (D) of EPSCs evoked by the second pulse ($eEPSC_2$) at P8–12.

Open symbols represent individual cells. Population data (closed symbols) are presented as mean ± SEM. ***P<0.001, **P<0.01, ns – not significant (simple contrasts). See also Table S1.

Table S1. Descriptive and inductive statistics, related to Figures 1–7 and S1–S3.

GLMM, generalized linear mixed model. ***P<0.001, **P<0.01, *P<0.05, ns–not significant.

#	Figure	Parameter	Genotype (Dataset)	Condition	Biological replicate (n) and number of animals	Mean ± SEM		Fixed effects (GLMM) (other test if specified)				Simple contrasts (other test if specifed)				
								Source	F	P	Sign.	Genotype	t	df	P	Sign.
1	1E	CaT frequency	hM4Di	Cont	5 slices	1.4 ± 0.4	min^{-1}	Model:	75.9	8.7E-07	***	hM4Di				
			hM4Di	C21	2 animals	0.0 ± 0.0	min^{-1}	Genotype:	29.7	4.1E-04	***	Cont vs. C21:	4.0	9.0	3.2E-03	**
			WT	Cont	6 slices	2.0 ± 0.4	min^{-1}	Condition:	143.4	7.8E-07	***	WT				
			WT	C21	3 animals	1.4 ± 0.3	min^{-1}	Int.:	100.8	3.5E-06	***	Cont vs. C21:	1.5	9.0	1.6E-01	ns
2	1F	GDP frequency	hM4Di	Cont	5 slices	2.2 ± 0.7	min^{-1}	Model:	14.3	1.8E-02	*	hM4Di				
			hM4Di	C21	2 animals	0.0 ± 0.0	min^{-1}	Genotype:	5.2	5.9E-02	ns	Cont vs. C21:	4.4	7.1	3.0E-03	**
			WT	Cont	6 slices	2.7 ± 0.5	min^{-1}	Condition:	16.4	4.8E-03	**	WT				
			WT	C21	3 animals	2.1 ± 0.3	min^{-1}	Int.:	6.2	4.2E-02	*	Cont vs. C21:	1.2	7.1	2.8E-01	ns
3	2B	RMP	hM4Di	Cont	10 cells	-65.3 ± 1.8	mV	Model:	3.1	5.4E-02	ns					
			hM4Di	C21	3 animals	-66.2 ± 1.7	mV	Genotype:	8.0	1.3E-02	NA					
			WT	Cont	7 cells	-59.0 ± 1.6	mV	Condition:	0.1	7.2E-01	NA					
			WT	C21	6 animals	-58.6 ± 1.9	mV	Int.:	1.0	3.4E-01	NA					
4	2C	R_m	hM4Di	Cont	10 cells	649.8 ± 118.7	MΩ	Model:	4.5	1.0E-02	*					
			hM4Di	C21	3 animals	539.9 ± 84.8	MΩ	Genotype:	0.2	6.5E-01	ns					
			WT	Cont	7 cells	602.2 ± 20.8	MΩ	Condition:	10.5	2.9E-03	**					
			WT	C21	6 animals	562.5 ± 38.1	MΩ	Int.:	1.1	3.0E-01	ns					
5	2E	RMP	hM4Di	Cont	10 cells	-60.4 ± 2.1	mV	Model:	0.031	9.9E-01	ns					
			hM4Di	C21	6 animals	-60.5 ± 2.5	mV	Genotype:	0.0	8.3E-01	NA					
			WT	Cont	9 cells	-61.1 ± 1.3	mV	Condition:	0.0	9.9E-01	NA					
			WT	C21	3 animals	-61.0 ± 1.4	mV	Int.:	0.0	8.8E-01	NA					
6	2F	R_m	hM4Di	Cont	10 cells	276.4 ± 38.8	MΩ	Model:	0.51	6.8E-01	ns					
			hM4Di	C21	6 animals	268.0 ± 32.9	MΩ	Genotype:	0.1	7.5E-01	NA					
			WT	Cont	9 cells	249.0 ± 22.8	MΩ	Condition:	0.1	7.3E-01	NA					
			WT	C21	3 animals	265.3 ± 37.1	MΩ	Int.:	1.2	2.9E-01	NA					
7	3D	Max. inst. frequency	hM4Di	Cont	10 cells	43.0 ± 6.1	Hz	Model:	2.7	7.8E-02	ns					
			hM4Di	C21	3 animals	43.5 ± 5.0	Hz	Genotype:	2.5	1.3E-01	NA					
			WT	Cont	7 cells	59.4 ± 3.8	Hz	Condition:	2.8	1.1E-01	NA					
			WT	C21	6 animals	51.9 ± 6.6	Hz	Int.:	3.6	7.6E-02	NA					
8	3E	Max. mean frequency	hM4Di	Cont	10 cells	14.0 ± 1.6	Hz	Model:	8.3	1.6E-03	**					
			hM4Di	C21	3 animals	12.5 ± 1.3	Hz	Genotype:	0.0	9.8E-01	ns					
			WT	Cont	7 cells	15.2 ± 1.7	Hz	Condition:	22.9	2.8E-04	***					
			WT	C21	6 animals	11.4 ± 1.7	Hz	Int.:	4.5	5.2E-02	ns					
9	3F	AP threshold	hM4Di	Cont	10 cells	-33.0 ± 0.6	mV	Model:	1.1	4.6E-01	ns					
			hM4Di	C21	3 animals	-32.5 ± 0.8	mV	Genotype:	2.4	1.4E-01	NA					
			WT	Cont	7 cells	-29.4 ± 2.4	mV	Condition:	0.5	5.2E-01	NA					
			WT	C21	6 animals	-29.0 ± 2.8	mV	Int.:	0.0	9.0E-01	NA					
10	3G	Rheobase	hM4Di	Cont	10 cells	51.0 ± 8.7	pA	Model:	0.89	4.7E-01	ns					
			hM4Di	C21	3 animals	51.0 ± 7.2	pA	Genotype:	0.4	5.4E-01	NA					
			WT	Cont	7 cells	42.9 ± 2.9	pA	Condition:	1.5	2.5E-01	NA					
			WT	C21	6 animals	47.1 ± 4.7	pA	Int.:	1.5	2.5E-01	NA					
11	3K	Max. inst. frequency	hM4Di	Cont	10 cells	116.2 ± 11.6	Hz	Model:	1.4	2.9E-01	ns					
			hM4Di	C21	6 animals	110.7 ± 9.6	Hz	Genotype:	2.4	1.4E-01	NA					
			WT	Cont	9 cells	135.2 ± 9.7	Hz	Condition:	0.3	5.7E-01	NA					
			WT	C21	3 animals	136.7 ± 11.3	Hz	Int.:	1.0	3.2E-01	NA					
12	3L	Max. mean frequency	hM4Di	Cont	10 cells	31.5 ± 2.0	Hz	Model:	2.8	8.2E-02	ns					
			hM4Di	C21	6 animals	30.8 ± 2.6	Hz	Genotype:	5.1	3.9E-02	NA					
			WT	Cont	9 cells	39.6 ± 2.1	Hz	Condition:	1.2	3.0E-01	NA					
			WT	C21	3 animals	37.6 ± 3.0	Hz	Int.:	0.2	6.3E-01	NA					
13	3M	AP threshold	hM4Di	Cont	10 cells	-39.8 ± 2.1	mV	Model:	2.3	9.9E-02	ns					
			hM4Di	C21	6 animals	-40.0 ± 2.1	mV	Genotype:	1.1	3.1E-01	NA					
			WT	Cont	9 cells	-42.1 ± 1.3	mV	Condition:	4.0	5.4E-02	NA					
			WT	C21	3 animals	-43.1 ± 1.6	mV	Int.:	2.0	1.7E-01	NA					
14	3N	Rheobase	hM4Di	Cont	10 cells	94.0 ± 9.0	pA	Model:	0.93	4.5E-01	ns					
			hM4Di	C21	6 animals	96.0 ± 8.3	pA	Genotype:	1.8	2.0E-01	NA					
			WT	Cont	9 cells	80.0 ± 8.8	pA	Condition:	0.0	9.7E-01	NA					
			WT	C21	3 animals	77.8 ± 8.5	pA	Int.:	0.6	4.4E-01	NA					
15	4C	mEPSC frequency	hM4Di	Cont	12 cells	0.13 ± 0.03	Hz	Model:	8.7	7.3E-04	***	hM4Di				
			hM4Di	C21	8 animals	0.05 ± 0.01	Hz	Genotype:	2.8	1.1E-01	ns	Cont vs. C21:	3.9	18	1.1E-03	**
			WT	Cont	8 cells	0.10 ± 0.01	Hz	Condition:	2.5	1.3E-01	ns	WT				
			WT	C21	3 animals	0.16 ± 0.06	Hz	Int.:	19.3	3.5E-04	***	Cont vs. C21:	-1.6	18	1.4E-01	ns
16	4E	mEPSC amplitude	hM4Di	Cont	12 cells	21.8 ± 0.7	pA	Model:	1.7	2.1E-01	ns					
			hM4Di	C21	8 animals	23.4 ± 1.8	pA	Genotype:	0.2	6.7E-01	NA					
			WT	Cont	8 cells	26.1 ± 3.5	pA	Condition:	1.2	2.9E-01	NA					
			WT	C21	3 animals	21.2 ± 2.1	pA	Int.:	4.4	5.0E-02	NA					
17	4H	mEPSC frequency	hM4Di	Cont	13 cells	0.31 ± 0.07	Hz	Model:	15.0	1.3E-05	***	hM4Di				
			hM4Di	C21	8 animals	0.14 ± 0.02	Hz	Genotype:	1.1	3.0E-01	ns	Cont vs. C21:	4.5	21	1.7E-04	***
			WT	Cont	10 cells	0.27 ± 0.05	Hz	Condition:	22.9	1.0E-04	***	WT				
			WT	C21	3 animals	0.24 ± 0.03	Hz	Int.:	15.1	8.5E-04	***	Cont vs. C21:	0.59	21	5.6E-01	ns

Table S1 (continued)

18	4J	mEPSC amplitude	hM4Di	Cont	13	cells	25.6 ± 1.5	pA	Model:	3.6	3.0E-02	*					
			hM4Di	C21	8	animals	25.7 ± 2.1	pA	Genotype:	4.8	4.0E-02	*					
			WT	Cont	10	cells	22.8 ± 1.6	pA	Condition:	4.8	3.9E-02	*					
			WT	C21	3	animals	19.1 ± 1.2	pA	Int.:	4.1	5.6E-02	ns					
19	5B	$eEPSC_1$ amplitude	hM4Di	Cont	8	cells	34.1 ± 8.5	pA	Model:	70.0	1.4E-12	***	hM4Di				
			hM4Di	C21	3	animals	1.7 ± 1.3	pA	Dataset:	12.9	1.3E-03	**	Cont vs. C21:	3.3	26	3.1E-03	**
			hM4Di	Cont	7	cells	36.9 ± 8.6	pA	Condition:	111.7	6.6E-11	***	hM4Di				
			hM4Di	Veh.	4	animals	23.7 ± 4.0	pA	Int.:	72.6	5.3E-09	***	Cont vs. Veh.:	1.3	26	2.1E-01	ns
20	5C	$eEPSC_1$ failure rate	hM4Di	Cont	8	cells	0.25 ± 0.06		Model:	82.3	4.9E-09	***	hM4Di				
			hM4Di	C21	3	animals	0.92 ± 0.04		Dataset:	15.9	1.5E-03	**	Cont vs. C21:	-15.1	13	1.3E-09	***
			hM4Di	Cont	7	cells	0.25 ± 0.07		Condition:	121.9	5.6E-08	***	hM4Di				
			hM4Di	Veh.	4	animals	0.29 ± 0.08		Int.:	91.9	2.9E-07	***	Cont vs. Veh.:	-0.99	13	3.4E-01	ns
21	5E	$eEPSC_1$ amplitude	hM4Di	Cont	9	cells	35.2 ± 7.0	pA	Model:	42.4	6.7E-11	***	hM4Di				
			hM4Di	C21	3	animals	2.5 ± 1.7	pA	Dataset:	20.3	9.4E-05	***	Cont vs. C21:	4.0	30	3.5E-04	***
			hM4Di	Cont	8	cells	38.2 ± 6.4	pA	Condition:	57.1	2.0E-08	***	hM4Di				
			hM4Di	Veh.	4	animals	31.6 ± 4.7	pA	Int.:	43.4	2.7E-07	***	Cont vs. Veh.:	0.66	30	5.2E-01	ns
22	5F	$eEPSC_1$ failure rate	hM4Di	Cont	9	cells	0.22 ± 0.06		Model:	77.6	9.0E-10	***	hM4Di				
			hM4Di	C21	3	animals	0.91 ± 0.05		Dataset:	65.2	7.7E-07	***	Cont vs. C21:	-12.2	15	3.4E-09	***
			hM4Di	Cont	8	cells	0.14 ± 0.04		Condition:	71.5	4.3E-07	***	hM4Di				
			hM4Di	Veh.	4	animals	0.15 ± 0.03		Int.:	68.9	5.4E-07	***	Cont vs. Veh.:	-0.11	15	9.2E-01	ns
23	6D	Normalized bandpower (8-40Hz)	hM4Di	Cont	5	animals	5.4 ± 0.9		Model:	27.6	3.2E-09	***	hM4Di				
			hM4Di	C21			0.9 ± 0.0		Dataset:	10.9	4.4E-04	***	Cont vs. C21:	3.2	24	3.6E-03	**
			WT	Cont	5	animals	8.9 ± 5.2		Condition:	15.3	6.6E-04	***	WT				
			WT	C21			9.8 ± 5.7		Int.:	50.6	2.5E-09	***	Cont vs. C21:	-0.57	24	5.8E-01	ns
			hM4Di	Cont	5	animals	12.1 ± 2.5						hM4Di				
			hM4Di	Veh.			20.6 ± 6.0						Cont vs. Veh.:	-2.3	24	3.4E-02	*
24	6E	eSPW occurrence	hM4Di	Cont	5	animals	2.1 ± 0.3	min^{-1}	Model:	14.5	1.4E-06	***	hM4Di				
			hM4Di	C21			0.0 ± 0.0	min^{-1}	Dataset:	8.5	1.6E-03	**	Cont vs. C21:	6.7	24	5.9E-07	***
			WT	Cont	5	animals	2.3 ± 0.4	min^{-1}	Condition:	21.3	1.1E-04	***	WT				
			WT	C21			1.4 ± 0.4	min^{-1}	Int.:	17.1	2.4E-05	***	Cont vs. C21:	2.8	24	1.0E-02	**
			hM4Di	Cont	5	animals	2.1 ± 0.1	min^{-1}					hM4Di				
			hM4Di	Veh.			2.5 ± 0.2	min^{-1}					Cont vs. Veh.:	-1.54	24	1.4E-01	ns
25	7D	Normalized bandpower (8-40Hz)	hM4Di	Cont	5	animals	39.8 ± 7.8		Model:	60.9	7.6E-13	***	hM4Di				
			hM4Di	C21			2.5 ± 0.6		Dataset:	14.8	6.5E-05	***	Cont vs. C21:	2.6	24	1.4E-02	*
			WT	Cont	5	animals	57.6 ± 20.4		Condition:	73.4	9.1E-09	***	WT				
			WT	C21			61.9 ± 22.0		Int.:	100.7	2.1E-12	***	Cont vs. C21:	-0.20	24	8.4E-01	ns
			hM4Di	Cont	5	animals	185.2 ± 69.1						hM4Di				
			hM4Di	Veh.			267.1 ± 116.9						Cont vs. Veh.:	-1.3	24	2.1E-01	ns
26	7E	LFP burst occurrence	hM4Di	Cont	5	animals	4.0 ± 0.4	min^{-1}	Model:	5.7	1.3E-03	**	hM4Di				
			hM4Di	C21			2.0 ± 0.2	min^{-1}	Dataset:	5.3	1.2E-02	*	Cont vs. C21:	3.8	24	7.8E-04	***
			WT	Cont	5	animals	3.9 ± 0.5	min^{-1}	Condition:	3.5	7.4E-02	ns	WT				
			WT	C21			4.7 ± 0.5	min^{-1}	Int.:	7.2	3.5E-03	**	Cont vs. C21:	-1.52	24	1.4E-01	ns
			hM4Di	Cont	5	animals	3.1 ± 0.4	min^{-1}					hM4Di				
			hM4Di	Veh.			2.6 ± 0.5	min^{-1}					Cont vs. Veh.:	0.9	24	3.8E-01	ns
27	S1C	RMP	WT	Cont	6	cells	-61.3 ± 1.5	mV	N/A				WT				
			WT	Baclofen	2	animals	-67.2 ± 1.2	mV					(Paired t-test)	3.6	5	1.5E-02	*
28	S1E	Rm	WT	Cont	6	cells	154.5 ± 20.2	mV	N/A				WT				
			WT	Baclofen	2	animals	85.8 ± 7.7	mV					(Paired t-test)	4.9	5	4.6E-03	**
29	S2B	RMP	hM4Di	Cont	6	cells	-60.5 ± 1.4	mV		0.1	7.3E-01	ns					
			hM4Di	C21 (#1)	1	animal	-60.8 ± 1.7	mV	(1W-RM-ANOVA. Huynh-Feldt correction)								
			hM4Di	C21 (#2)			-60.4 ± 2.3	mV									
30	S2D	RMP	hM4Di	Cont	6	cells	-60.7 ± 1.2	mV		0.3	6.8E-01	ns					
			hM4Di	C21 (#1)	1	animal	-60.6 ± 1.3	mV	(1W-RM-ANOVA. Huynh-Feldt correction)								
			hM4Di	C21 (#2)			-60.3 ± 1.7	mV									
			hM4Di	C21 (#3)			-60.6 ± 1.8	mV									
31	S3A	$eEPSC_1$ amplitude	hM4Di	Trial #01	7	cells	-29.6 ± 14.2	pA	Model:	0.4	5.2E-01	ns					
			hM4Di	Trial #02	4	animals	-28.3 ± 12.6	pA	Condition:	0.4	5.2E-01	NA					
			hM4Di	Trial #03			-39.2 ± 11.1	pA	(Trial)								
			hM4Di	Trial #04			-26.2 ± 11.7	pA									
			hM4Di	Trial #05			-33.6 ± 13.8	pA									
			hM4Di	Trial #06			-44.7 ± 24.0	pA									
			hM4Di	Trial #07			-37.6 ± 8.3	pA									
			hM4Di	Trial #08			-21.3 ± 7.1	pA									
			hM4Di	Trial #09			-29.9 ± 12.4	pA									
			hM4Di	Trial #10			-51.2 ± 8.6	pA									
			hM4Di	Trial #11			-37.4 ± 13.8	pA									
			hM4Di	Trial #12			-35.1 ± 15.6	pA									
			hM4Di	Trial #13			-35.1 ± 14.1	pA									
			hM4Di	Trial #14			-44.3 ± 23.8	pA									
			hM4Di	Trial #15			-33.3 ± 12.2	pA									
			hM4Di	Trial #16			-41.3 ± 10.7	pA									
			hM4Di	Trial #17			-36.4 ± 9.3	pA									
			hM4Di	Trial #18			-42.8 ± 14.1	pA									
			hM4Di	Trial #19			-30.4 ± 9.9	pA									
			hM4Di	Trial #20			-32.3 ± 11.8	pA									
			hM4Di	Trial #21			-35.8 ± 15.7	pA									
			hM4Di	Trial #22			-29.1 ± 13.7	pA									
			hM4Di	Trial #23			-54.5 ± 24.3	pA									
			hM4Di	Trial #24			-45.9 ± 20.4	pA									
			hM4Di	Trial #25			-25.5 ± 10.9	pA									
			hM4Di	Trial #26			-42.9 ± 22.0	pA									
			hM4Di	Trial #27			-33.1 ± 21.8	pA									
			hM4Di	Trial #28			-46.5 ± 13.1	pA									
			hM4Di	Trial #29			-50.7 ± 16.2	pA									
			hM4Di	Trial #30			-34.6 ± 11.4	pA									

Table S1 (continued)

32	S3A	$eEPSC_1$ amplitude	hM4Di	Trial #01	8	cells	-44.1 ± 8.7	pA	Model:	1.1	3.0E-01	ns					
			hM4Di	Trial #02	4	animals	-24.6 ± 8.6	pA	Condition:	1.1	3.0E-01	NA					
			hM4Di	Trial #03			-35.6 ± 12.6	pA	(Trial)								
			hM4Di	Trial #04			-46.1 ± 12.3	pA									
			hM4Di	Trial #05			-23.1 ± 9.7	pA									
			hM4Di	Trial #06			-28.7 ± 9.0	pA									
			hM4Di	Trial #07			-45.4 ± 12.9	pA									
			hM4Di	Trial #08			-35.9 ± 9.8	pA									
			hM4Di	Trial #09			-29.7 ± 6.7	pA									
			hM4Di	Trial #10			-45.9 ± 10.8	pA									
			hM4Di	Trial #11			-48.4 ± 14.7	pA									
			hM4Di	Trial #12			-48.4 ± 15.3	pA									
			hM4Di	Trial #13			-60.1 ± 8.5	pA									
			hM4Di	Trial #14			-32.1 ± 8.3	pA									
			hM4Di	Trial #15			-50.1 ± 17.0	pA									
			hM4Di	Trial #16			-40.9 ± 14.2	pA									
			hM4Di	Trial #17			-36.6 ± 13.0	pA									
			hM4Di	Trial #18			-41.8 ± 19.0	pA									
			hM4Di	Trial #19			-56.1 ± 14.3	pA									
			hM4Di	Trial #20			-45.1 ± 15.2	pA									
			hM4Di	Trial #21			-39.8 ± 12.3	pA									
			hM4Di	Trial #22			-43.5 ± 13.5	pA									
			hM4Di	Trial #23			-41.7 ± 13.5	pA									
			hM4Di	Trial #24			-30.7 ± 6.3	pA									
			hM4Di	Trial #25			-34.9 ± 14.6	pA									
			hM4Di	Trial #26			-45.7 ± 11.5	pA									
			hM4Di	Trial #27			-38.3 ± 12.6	pA									
			hM4Di	Trial #28			-30.6 ± 10.3	pA									
			hM4Di	Trial #29			-26.5 ± 8.7	pA									
			hM4Di	Trial #30			-22.5 ± 4.8	pA									
33	S3C	$eEPSC_2$ amplitude	hM4Di	Cont	8	cells	-55.7 ± 12.7	pA	Model:	48.1	9.4E-11	***	hM4Di				
			hM4Di	C21	3	animals	-4.0 ± 2.0	pA	Dataset:	9.8	4.2E-03	**	Cont vs. C21:	3.3	26	2.6E-03	**
			hM4Di	Cont	7	cells	-58.4 ± 16.7	pA	Condition:	70.8	6.7E-09	***	hM4Di				
			hM4Di	Veh.	4	animals	-44.9 ± 9.7	pA	Int.:	54.8	7.4E-08	***	Cont vs. Veh.:	0.7	26	5.0E-01	ns
34	S3D	$eEPSC_2$ failure rate	hM4Di	Cont	8	cells	0.08 ± 0.03		Model:	129.4	1.0E-15	***	hM4Di				
			hM4Di	C21	3	animals	0.85 ± 0.06		Dataset:	43.0	5.9E-07	***	Cont vs. C21:	-18.6	26	2.2E-16	***
			hM4Di	Cont	7	cells	0.08 ± 0.03		Condition:	176.8	4.2E-13	***	hM4Di				
			hM4Di	Veh.	4	animals	0.11 ± 0.05		Int.:	145.5	3.7E-12	***	Cont vs. Veh.:	-0.85	26	4.0E-01	ns
35	S3E	$eEPSC_2$ amplitude	hM4Di	Cont	9	cells	-55.3 ± 11.3	pA	Model:	47.2	1.8E-11	***	hM4Di				
			hM4Di	C21	3	animals	-4.9 ± 2.4	pA	Dataset:	19.9	1.1E-04	***	Cont vs. C21:	4.6	30	6.5E-05	***
			hM4Di	Cont	8	cells	-51.1 ± 5.8	pA	Condition:	66.8	4.0E-09	***	hM4Di				
			hM4Di	Veh.	4	animals	-41.6 ± 5.4	pA	Int.:	47.8	1.1E-07	***	Cont vs. Veh.:	0.9	30	4.0E-01	ns
36	S3F	$eEPSC_2$ failure rate	hM4Di	Cont	9	cells	0.09 ± 0.05		Model:	59.5	2.9E-12	***	hM4Di				
			hM4Di	C21	3	animals	0.81 ± 0.07		Dataset:	55.0	4.5E-08	***	Cont vs. C21:	-11.1	28	9.0E-12	***
			hM4Di	Cont	8	cells	0.05 ± 0.03		Condition:	48.6	1.4E-07	***	hM4Di				
			hM4Di	Veh.	4	animals	0.03 ± 0.01		Int.:	59.7	2.1E-08	***	Cont vs. Veh.:	0.50	28	6.2E-01	ns
